# Low Molecular Weight Procyanidins from Grape Seeds Enhance the Impact of 5-Fluorouracil Chemotherapy on *Caco-2* Human Colon Cancer Cells

**DOI:** 10.1371/journal.pone.0098921

**Published:** 2014-06-06

**Authors:** Ker Y. Cheah, Gordon S. Howarth, Keren A. Bindon, James A. Kennedy, Susan E. P. Bastian

**Affiliations:** 1 Wine Science and Business Group, School of Agriculture, Food and Wine, Waite Campus, The University of Adelaide, PMB 1, Glen Osmond, Australia; 2 Centre for Paediatric and Adolescent Gastroenterology, Children, Youth and Women's Health Service, North Adelaide, Australia; 3 School of Animal and Veterinary Sciences, Roseworthy Campus, The University of Adelaide, Australia; 4 The Australian Wine Research Institute, Urrbrae, Adelaide, Australia; 5 Department of Viticulture and Enology, California State University, Fresno, California, United States of America; University of Alabama at Birmingham, United States of America

## Abstract

**Objective:**

Grape seed procyanidins (PC) are flavan-3-ol oligomers and polymers known for their biological activity in the gut. Grape seed extract (GSE) have been reported to reduce intestinal injury in a rat model of mucositis. We sought to investigate effects of purified PC fractions differing in mean degree of polymerization (mDP) combined with 5-Fluorouracil (5-FU) chemotherapy on the viability of colon cancer cells (Caco-2).

**Design:**

SixPC fractions (F1-F6) were isolated from Cabernet Sauvignon seeds at two ripeness stages: pre-veraison unripe (immature) and ripe (mature), utilizing step gradient, low-pressure chromatography on a Sephadex LH-20 resin. Fractions were tested on Caco-2 cells, alone and in combination with 5-FU. Eluted fractions were characterized by phloroglucinolysis and gel permeation chromatography. Cell viability was determined by the 3-(4,5-Dimethylthiazol-2yl)-2,5-diphenyl-tetrazolium bromide) (MTT) assay.

**Results:**

All isolated fractions significantly reduced Caco-2 cell viability compared to the control (P<0.05), but F2 and F3 (mDP 2–6) were the most active fractions (immature F2 = 32% mDP 2.4, F3 = 35% mDP 5.8 and mature F2 = 13% mDP 3.6 and F3 = 17% mDP 5.9; percentage of viable cells remaining) on Caco-2 cells. When combined with 5-FU, immature fractions F1-F3 enhanced the cell toxicity effects of 5-FU by 27–73% (*P<*0.05). Mature seed PC fractions (F1–F4) significantly enhanced the toxicity of 5-FU by 60–83% against Caco-2 cells (*P<*0.05). Moreover, some fractions alone were more potent at decreasing viability in Caco-2 cells (*P<*0.05; immature fractions = 65–68% and mature fractions = 83–87%) compared to 5-FU alone (37%).

**Conclusions:**

PCs of mDP 2–6 (immature F1-F3 and mature F1 and F4)not only enhanced the impact of 5-FU in killing Caco-2 cells, but also surpassed standard 5-FU chemotherapy as an anti-cancer agent.The bioactivity of PC is therefore attributed primarily to lower molecular weight PCs.

## Introduction

Colorectal cancer has the second highest mortality, and is the fourth most frequently diagnosed form of cancer in the United States [1]. The primary treatment for colon cancer involves surgical bowel resection and chemotherapy, most often by 5-Fluorouracil (5-FU) [2]. Unfortunately, chemotherapy cannot discriminate between normal and cancer cells, and it targets areas where cells are replaced at a high rate, such as in the mouth and gut [3]. This leads to the development of mucositis (gastrointestinal toxicity). Current mucositis treatments are largely ineffective as they target only the symptoms, but not the pathogenesis of the condition [3]. Thus, it is important to seek new alternative treatments which not only target mucositis but also enhance chemotherapeutic action without compromising the well-being of patients.

Increasingly, grape seed extracts (GSEs) are being studied due to their reported health benefits for a variety of disorders such as cancer [4],cardiovascular disease[5], [6] and ulcerative colitis [7], [8]. The beneficial effects of GSEs have been attributed to polyphenolic procyanidins (PCs) [9], comprising flavan-3-ol subunits. The polymer length of PC is described by the degree of polymerization (DP). Due to their polymerized structure, cellular absorption is restricted to oligomers with a lower DP, leaving the larger DP molecules for adsorption in the gut lumen following oral administration [10], [11]. A number of studies have reported the polymerization and galloylation of PCs to be responsible for their anti-proliferative effects on transformed cells [9], [12], [13].

There is increasing evidence suggesting GSEs are effective at reducing the proliferation of cancer cells without being cytotoxic to normal cells [14]. Additionally, GSE has demonstrated partial amelioration of intestinal damage in a rat model of intestinal mucositis and has reduced gastrointestinal cell toxicity following chemotherapy treatment in normal IEC-6 intestinal cells [15]. However, the bioactive components in GSE remain unknown. The primary aim of the current study was to identify the chemical composition of the bioactive PC fractions, and investigate their potential in combination with 5-FU chemotherapy, for their effects on the viability of colon cancer cells. When in combination with 5-FU, certain PC fractions further enhanced toxicity in colon cancer cells.

## Materials and Methods

### Ethic statements

Cabernet Sauvignon grape samples were obtained from commercial vineyards, Accolade Wines (Orlando) in the Langhorne Creek growing region of South Australia, which has a latitude 35°16′11.56″S and a longitude 139°00′14.47″E, with an elevation approximately 28 m above sea level. No permits were required for the described study, which complied with all relevant regulations.

### Grape sampling and preparation

Grape samples were collected at different stages of ripeness: immature (preveraison, green seeds) and mature (25–26° Brix, brown seeds) during the 2009 growing season. Grape berries were prepared according to a previously described method [16] in which grapes were collected and kept frozen at −20°C. While still frozen, seeds were removed from the flesh with a scalpel. Seeds were then wiped clean of flesh with a paper towel and re-frozen at −20°C prior to extraction.

### Preparation and extraction of seed procyanidins (PCs)

PC extraction commenced by extracting seeds (100 g) overnight for 18 h in 70% acetone (200 mL, v/v) and ascorbic acid (1 g/L). The extract was concentrated under reduced pressure at 35°C (HeidolphLaborota 4011 rotary evaporator, John Morris Scientific, Adelaide, Australia), and lyophilized to a dry powder (Dynavac FD3 freeze drier, Dynavac Pty Ltd, Sydney, Australia). Yields for each samples were: immature 6.31 g and mature 10.29 g. PC seed powder (5 g) was dissolved in 50 mL of methanol (60%, v/v) containing trifluoroacetic acid (TFA) (0.05%, v/v) and then applied (∼18.3 mL/min) to a 300 mm x 21 mm glass column (Michel-Miller, Vineland, NJ, USA) containing Sephadex LH20 chromatography resin (Amersham, Uppsala, Sweden) to an approximate bed volume of 93 mL, and washed off in 250 mL of methanol (60%, v/v) containing TFA (0.05%, v/v) to remove low molecular weight monomers. PC was then recovered in 150 mL of aqueous acetone (70%, v/v) and the extracts was concentrated in a rotary evaporator at 35°C to remove acetone. The aqueous solution was extracted with hexane to remove residual lipophilic material using a separatory funnel. The aqueous extract was freeze-dried into powder and recovered amount for two extracts were; immature, 2.5 g and mature, 0.9 g respectively. Powders were kept under nitrogen and at −20°C prior to fractionation.

For fractionation of PCs, seed PC powder (0.5 g) was dissolved in methanol (60%, v/v) containing TFA (0.05%, v/v) and applied to the same column under identical conditions. PC seed extract was fractionated according to a step-gradient elution method described previously [16] to produce 6 fractions with increasing mean degree of polymerization (mDP) and molecular weights of PCs designated as F1 to F6. The eluted fractions were concentrated under pressure at 35°C to remove organic solvents and further lyophilized into dry powder. The isolated fractions were stored at −20°C prior to analysis. Grape seed extract was a generous gift from Tarac Technologies (GraPex seed tannin; North Adelaide, South Australia) and was included in the study as a control. GSE (1 g) was dissolved in methanol (60%, v/v) containing TFA (0.05%, v/v) and fractionated accordingly.

### Acid catalysis of PC in the presence of excess phloroglucinol (Phloroglucinolysis)

Phloroglucinolysis was used to determine subunit composition, mDP and galloylation of PC. Pholoroglucinolysis was performed according to a previously described method [16]. Seed fractions were dissolved in methanol (10 mg/mL, v/v) and equal volumes (25 µL) of extract and phloroglucinol solution (0.2 N HCL and 100 g/L phloroglucinol and 20 g/L ascorbic acid) to give a final PC concentration of 5 g/L. The phloroglucinolysis reaction was carried out at 50°C for 25 min and analysed by RP-HPLC using (−)-epicatechin (Sigma Aldrich, St. Louis, MO) as the quantitative standard.

### Gel Permeation Chromatography (GPC)

Gel permeation chromatography was performed based on a previously described method [16]. The GPC technique characterises information on the size distribution of PC for each fraction. Fractionated samples (10 mg/mL) were dissolved in methanol and further diluted with 4 volumes of HPLC mobile phase (*N,N*-dimethylformamide containing glacial acetic acid (1%, v/v), water (5%, v/v) and 0.15 M lithium chloride). The flow-rate was maintained at 1 ml/min with a column temperature of 60°C and elution was monitored at 280 nm. The maximum amount of PC injected onto the column was 40 µg. PC seed fractions from a previous study[17] were used as standards for calibration. Calibration curves of fractionated PCs with their cumulative mass distribution were plotted and the mean molecular mass of the fractions was predicted at 50%.

### Ferric reducing antioxidant power (FRAP) assay

This assay was carried out following a modified protocol [18]. Briefly, FRAP reagent was prepared (300 mM acetate buffer, pH 3.6, 10 mM 2,4,6-tri[2-pyridyl]-s-triazine (TPTZ) solution in 40 mM HCL, 20 mM ferrous chloride; in 10∶1∶1 v/v) and kept in the dark at 37°C prior to analysis. Fractions were dissolved in dimethylsulfoxide (DMSO) (0.1 mg/mL) and 15 µL were added to 96-well plates. 15 µL of FRAP reagent was added to the wells containing the fractions and the plate was read at 593 nm after 4 min (Multiskan Spectrum, Therma Electron Corporation, Vantaa, Finland) using Skanit software 2.2. Ferrous sulphate (0.1–1 mM) was used to construct a standard curve and FRAP values of test compounds were expressed as mMFe(II)/g of sample. Each treatment was tested in triplicate and the whole experiment was repeated three times. The data are expressed as mean ± SEM of 3 independent experiments.

### Cell preparation and experimental treatment

The human colon cancer cell line, Caco-2 was obtained from American Type Culture Collection (ATCC, Manassas, USA). Cells were maintained in Dulbecco Modified Eagle Medium (DMEM) at 37°C and in a 5% CO_2_ incubator. Media were replaced twice in each week. PC fractions were dissolved in DMSO and kept at −20°C prior to analysis. Caco-2 cells were seeded at 1000 cells/well in 96-well tissue culture plates (Grenier Bio-one, Victoria, Australia) and incubated at 37°C in 5% CO_2_ for 48 h to allow attachment. After 48 h of incubation, the media were replaced with different concentrations of seed fractions dissolved in DMEM (µg/mL) containing <0.025% (v/v) of DMSO and 5-FU (100 µM) (DBL, Mayne Pharma Pty. Ltd., Victoria, Australia). Cells were further incubated at 37°C, 5% CO_2_ for 24, 48 and 72 hrs.

### MTT assay

The (3-(4,5-Dimethylthiazol-2yl)-2,5-diphenyl-tetrazolium bromide) (MTT) assay was performed based on a previously described method, with slight modification [19]. After exposure to the treatments (seed extracts and 5-FU), 50 µL of MTT (1 mg/mL in Dulbecco's Phosphate Buffered Saline) was added to each well and further incubated at 37°C, 5% CO_2_ for 4 h. After 4 h, the medium was aspirated and 100 µL of DMSO added to dissolve the formazan product. Plates were placed on a shaking incubator for 15 min and read at 570 nm by a UV spectrophotometer. Each treatment was tested in triplicate and the whole experiment was repeated three times. The data are expressed as mean ± SEM of 3 independent experiments. Data were expressed as number of viable cells compared with the percentage of control cells treated with serum free DMEM. Control cells represent either cell treated with serum free DMEM only or cell treated with 100 µM 5-FU.

### Statistical analysis

Each cell-based experiment was performed at least 3 times. Statistical analyses were performed using XLSTAT version 12.0 for Windows or PASW statistic version 18. Statistical analysis was determined by two-way ANOVA with Tukey's*post-hoc* test.

A Pearson's correlation coefficient was calculated to determine the relationship between cell viabilities, PC composition and antioxidant value. Statistical significance was considered at *P*<0.05.

### Chemicals

Acetone, ascorbic acid, methanol, trifluoroacetic acid (TFA), phloroglucinol, (−)-epicatechin, N,N-dimethylformamide, lithium chloride, sodium acetate (CH3COONa), 2,4,6-tri[2-pyridyl]-s-triazine (TPTZ), ferrous chloride and methanol were purchased from Sigma Chemical Co. Ltd, St Louis, MO. Hydrochloric acid (HCL), glacial acetic acid, Folin-ciocalteau reagent and ferrous sulphate were purchased from AnalaR, BDH, MERCK, Pty. Ltd., Australia. Tissue culture solutions include Dulbecco's Modified Eagle's Minimum Essential Medium (DMEM), Dulbecco's Phosphate Buffered Saline, dimethyl sulfoxide (DMSO) and 3-(4,5-Dimethylthiazol-2yl)-2,5-diphenyl-tetrazolium bromide) (MTT) were purchased from Gibco BRL, Life Tehnologies Pty Ltd, Australia.Pty. Ltd.

### Instrumentation

An Agilent model 1100 HPLC (Agilent Technologies Australia Pty Ltd., Melbourne, Australia) was used with Chemstation software for chromatographic analyses.

## Results

### Characterization of PC fractions

GSE was included in the current study as a control. The PC composition of GSE is illustrated in Table 1. Compared to Cabernet Sauvignon seed extracts, GSE had low mass conversion (23.8% w/w) and also a lower molecular mass, as measured by phloroglucinolysis and GPC. The PC terminal subunits in GSE were mostly dominated by (−)-epicatechin-3-*O*-gallate. Due to the apparently low contribution of PCs in GSE, we isolated PCs from fresh Cabernet Sauvignon seed harvested at different stages as previous reports showed a high mass conversion in unprocessed grape seeds [16].

**Table 1:** The chemical profiles of commercial grape seed extracts (GSE) and its isolated fractions characterized by phloroglucinolysis and gel permeation chromatography (GPC).

Procynidin	MC^a^	mDP^b^	galloylation	MM^c^	MM^d^	Mass^e^	Terminal subunits^f^			Extension subunits^f^		
Fraction	(%)		(%)	(subunit)	(GPC 50%)	(%)	C	E	ECG	C-P	E-P	ECG-P
GSE Control^g^	23.8	5.9	19	1871	1400	100	20.1	14.0	65.9	8.9	53.0	38.1
F1	37.1	2.0	6	593	974	7	43.7	40.3	16.0	19.1	63.4	17.5
F2	34.8	3.0	18	961	1870	8	22.3	16.9	60.8	11.6	59.8	28.6
F3	30.7	4.4	22	1423	3330	9	19.6	12.3	68.1	7.5	52.6	39.9
F4	14.4	6.5	23	2108	4490	11	19.6	10.3	70.2	7.6	47.3	45.1
F5	19.2	7.5	21	2432	4490	5	21.8	11.4	66.8	7.0	48.2	44.8
F6	28.7	9.1	21	2928	4400	5	22.4	10.6	67.1	6.3	48.9	44.8

a Mass conversion based on % recovery of procyanidin by phloroglucinolysis based on the gravimetric mass. ^b^Mean degree of polymerization in epicatechin units. ^c^Molecular mass as determined by phloroglucinolysis. ^d^Molecular mass as determined by GPC at 50% procyanidin elution. ^e^Percent composition of procyanidin fractions by gravimetric recovery. ^f^Percent composition of subunits (in moles) with the following subunit abbreviations: (-P), phloroglucinol adduct of extension subunit; C, (+)-catechin; EC, (−)-epicatechin; ECG, (−)-epicatechin-3-*O*-gallate. ^g^Crude grape seed extract of which 55% w/w eluted in F0 and 45% w/w was recovered as procyanidin for further fractionation.

Comparing purified PC from both seed samples, the immature seed had higher mass conversion (87%) compared to mature seeds (71%) (Table 2). The purified PCs were further fractionated into 6 fractions with increasing mDP or molecular mass. The mass conversion yield for conversion of PC into known PC was >49%, except for the highest mDP F6 (approximately 37%). The major differences between immature and mature seed fractions were that mature fractions had a higher proportion of (−)-epicatechin terminal subunits (9-30%) compared to the immature fractions (2-11%). Both mature and immature seed fractions showed a similar pattern of decreasing (−)-epicatechin terminal subunits with increasing mDP. Interestingly, the increases in polymerisation or molecular weight were mainly controlled by (−)-epicatechin-3-*O*-gallate extension subunits. The galloylation of immature fractions ranged from 15-35% and mature fractions range from 9–24%. Immature F2 had the highest galloylation (35%), mainly driven by a high proportion of (−)-epicatechin-3-*O*-gallate (88%).

**Table 2:** The chemical profile of immature and mature fractions characterized by phloroglucinolysis and gel permeation chromatography (GPC).

Procynidin	MC^a^	mDP^b^	galloylation	MM^c^	MM^d^	Mass^e^	Terminal subunits^f^			Extension subunits^f^		
Fraction	(%)		(%)	(subunit)	(GPC 50%)	(%)	C	E	ECG	C-P	E-P	ECG-P
Immature^g^	86.5	4.9	24	1593	3334	100	19.6	9.5	70.9	6.6	47.6	45.8
F1	48.7	2.0	15	619	882	7.7	35.8	11.0	53.1	22.7	65.0	12.3
F2	64.0	2.4	35	818	1280	12.9	9.6	2.2	88.3	12.4	61.1	26.0
F3	65.1	5.8	22	1893	5670	17.9	17.4	3.3	79.3	7.0	53.4	39.6
F4	62.7	10.0	23	3262	7600	24.4	17.5	4.1	78.4	5.8	47.9	46.3
F5	52.7	16.3	23	5313	7810	28.4	20.1	5.0	75.0	5.5	46.0	48.5
F6	37.7	18.8	22	6063	7940	8.8	24.4	4.6	71.0	5.4	47.3	47.3
Mature^g^	71.2	4.4	20	1404	2863	100	27	20.7	52.6	7.7	48.1	44.3
F1	64.0	2.3	9	706	896	10.9	36.8	30.2	32.9	20.0	62.1	17.9
F2	64.2	3.6	20	1150	1550	14.2	21.3	12.2	66.5	10.3	56.6	33.1
F3	66.0	5.9	23	1927	3620	21.3	19.5	9.3	71.1	6.7	49.0	44.4
F4	68.0	8.7	24	2840	6620	27.4	19.5	9.1	71.5	5.8	45.4	48.8
F5	54.1	12.0	23	3906	7190	17.6	21.1	9.3	69.6	5.9	45.9	48.2
F6	36.5	15.1	21	4886	7400	8.7	22.3	10.2	67.4	5.8	47.5	46.6

a Mass conversion based on % recovery of procyanidin by phloroglucinolysis based on the gravimetric mass. ^b^Mean degree of polymerization in epicatechin units. ^c^Molecular mass as determined by phloroglucinolysis. ^d^Molecular mass as determined by GPC at 50% procyanidin elution. ^e^Percent composition of procyanidin fractions by gravimetric recovery. ^f^Percent composition of subunits (in moles) with the following subunit abbreviations: (-P), phloroglucinol adduct of extension subunit; C, (+)-catechin; EC, (−)-epicatechin; ECG, (−)-epicatechin-3-*O*-gallate. ^g^Purifiedprocyanidin from immature (preveraison) and mature (ripe) Cabernet Sauvignon seeds prior to fractionation.

### Antioxidant capacity of seed fractions

**Figure 1:**
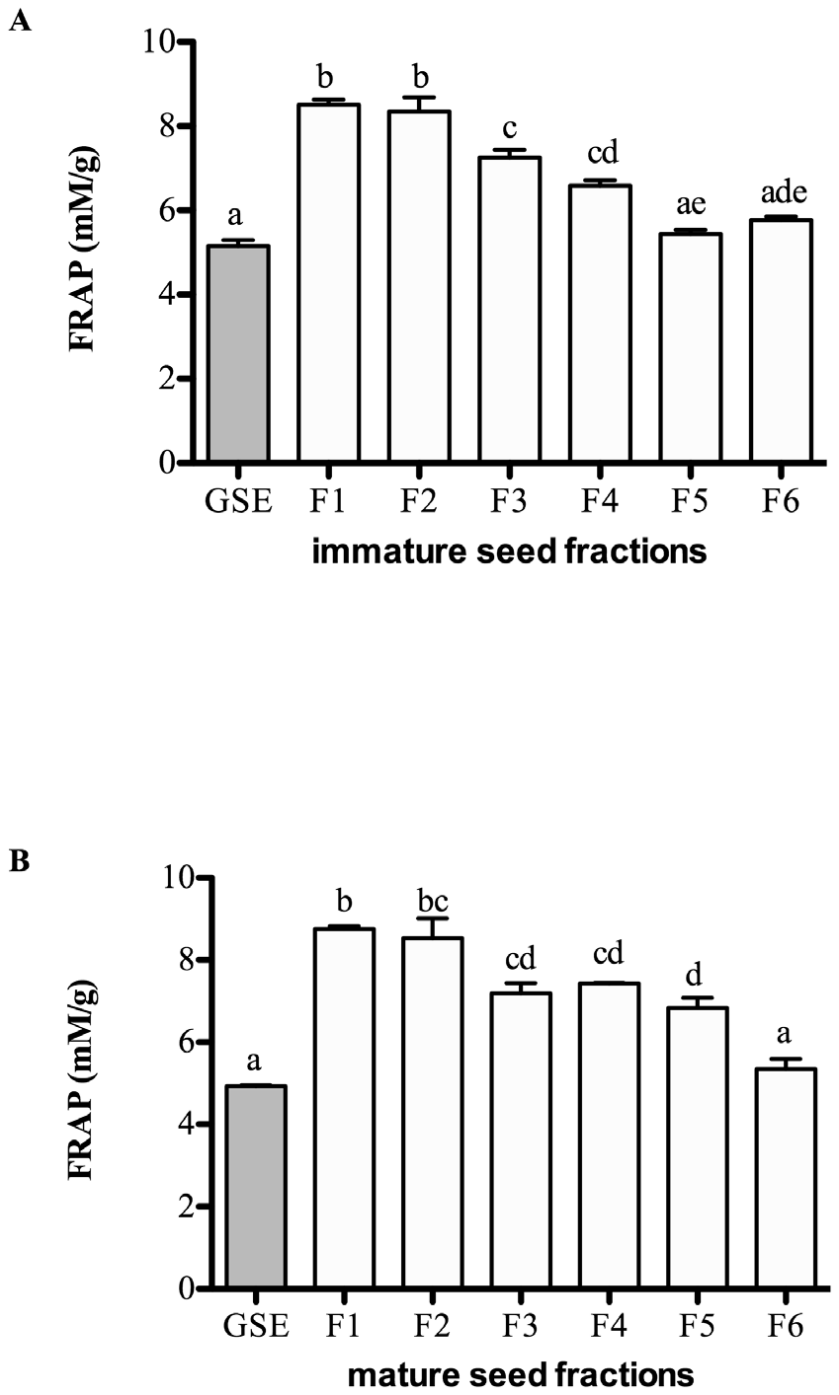
Antioxidant activity of the fractions from immature (A) and mature (B) seed extracts measured by Ferric reducing antioxidant power (FRAP) assay. Results are expressed as mean ± SEM of 3 independent experiments. Statistical analysis was determined by one-way ANOVA with Tukey's*post-hoc* test. Values with different letters (a,b,c) in each column are statistically different at P<0.05.

The antioxidant capacity of seed fractions was measured by the FRAP assay (Figure 1). GSE, which contained a mixture of oligomers and polymers of PCs, was included as a positive control to determine the antioxidant activity of the seed fractions. Compared to GSE (5 mM/g), all the fractions had higher FRAP values, ranging from 5.4–8.8 mM/g. The antioxidant capacity of the fractions decreased in the more polymerized PC fractions. The FRAP values were negatively correlated with mDP (r^2^ = −0.81, *P*<0.05).

### Effects of seed fractions on the viability of Caco-2 cells

**Figure 2:**
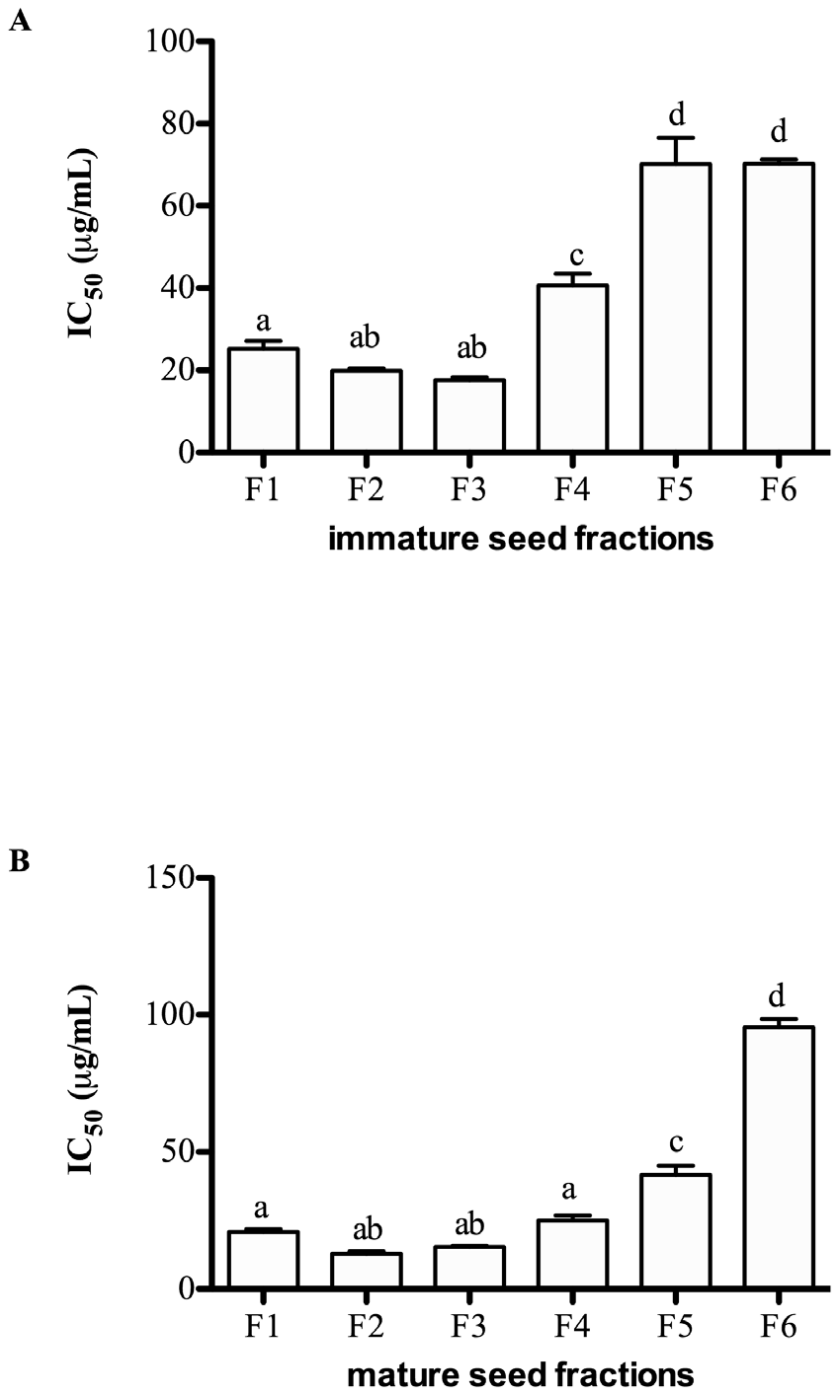
Viability of Caco-2 cells after exposure to fractions from immature (A) and mature (B) seed extracts for 72 hr measured by 3-(4,5-Dimethylthiazol-2yl)-2,5-diphenyl-tetrazolium bromide (MTT) assay. Data are expressed as IC_50_ or dose (µg/mL) inhibiting cell viability to 50% (mean ± SEM) of 3 independent experiments. Statistical analysis was determined by one-way ANOVA with Tukey's*post-hoc* test. Values with different letters (a,b,c) in each column were statistically different at P<0.05.

The cytotoxic effects of seed fractions on Caco-2 cells were determined by the MTT assay (Figure 2). Caco-2 cells were exposed to seed fractions for 72 hr and the data expressed as IC_50_, defined as the dose of each compound that inhibited cell viability to 50%, representing the mean value of three independent experiments. All fractions showed a degree of toxicity as indicated by decreased absorbance values. The IC_50_value (mean of the three independent experiments) for immature seed fractions (F1-F6) ranged from 17.7–70.2 µg/mL (Figure 2A). The number of viable cells was positively correlated with the mDP of immature seed fractions (r^2^ = 0.48, *P*<0.05) i.e., the fractions with highest mDP (F5 and F6) were less toxic to Caco-2 cells. Similar trends in terms of cytotoxic effects were also observed for the application of mature seed fractions (Figure 2B), where the number of viable cells was similarly correlated with the mDP of the fractions (r^2^ = 0.50, *P*<0.05). Mature seed fractions exhibited stronger cytotoxicity compared to immature seed fractions (Figure 2A and 2B).

### The combined effect of isolated seed fractions and 5-FU on the viability of Caco-2 cells

**Figure 3:**
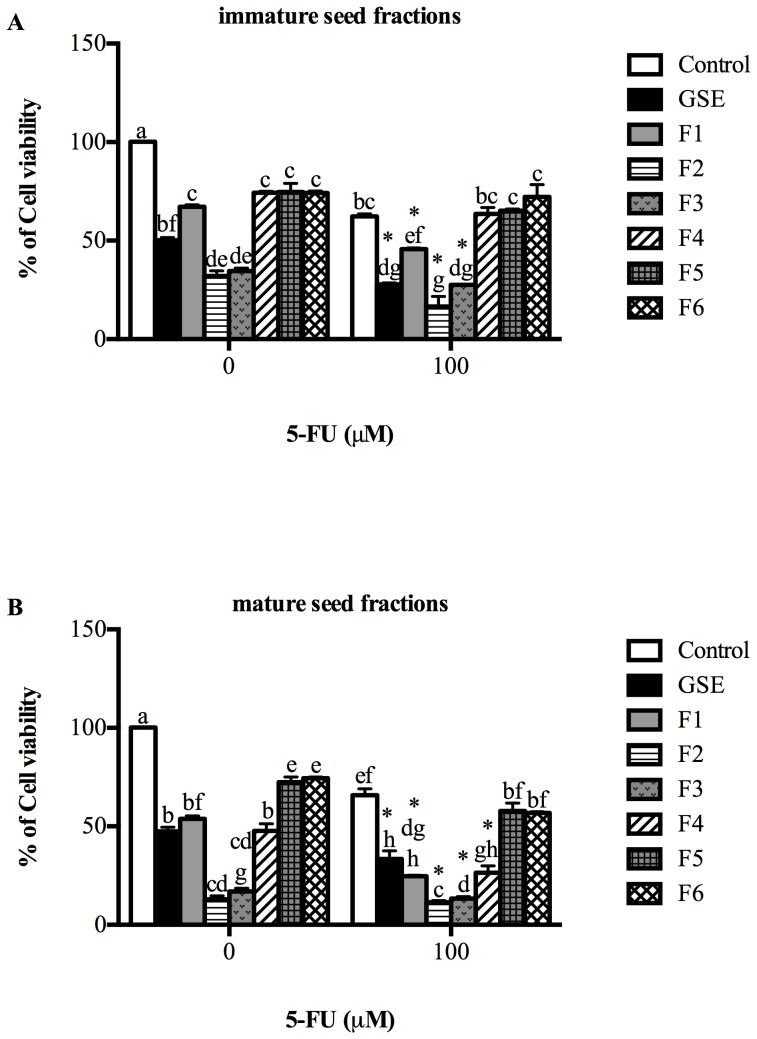
Combined effects of immature (A) and mature (B) fractions and 5-FU on Caco-2 cells for 72 hr measured by 3-(4,5-Dimethylthiazol-2yl)-2,5-diphenyl-tetrazolium bromide (MTT) assay. Data are presented as percentage of cell viability relative to viability of control cells. Data are expressed as mean ± SEM of 3 independent experiments. Statistical analysis was determined by two-way ANOVA with Tukey's*post-hoc* test. Values with different letters (a,b,c) in each column were statistically different at P<0.05. * indicates significant difference in 5-FU treated group when compared to 5-FU control.

The combined effect of seed fractions and 5-FU on Caco-2 cells was investigated (Figure 3). For immature seed fractions, the viability of Caco-2 cells was significantly inhibited by all seed fractions (Figure 3A). Compared to GSE (67%), F2 and F3 of immature seed extracts were more toxic to Caco-2 cells (F2, 32% and F3, 35% of control value, *P*<0.05). However, when seed fractions were present with 5-FU (100 uM), the growth inhibitory effects of 5-FU were significantly enhanced (*P*<0.05) (Figure 3A). 5-FU significantly reduced cell viability to 62% of control values (*P*<0.05). F1-F3 significantly enhanced the growth inhibitory activity of 5-FU (27%, 73% and 56% respectively compared to 5-FU control; *P*<0.05). F2 could therefore be considered a more potent chemotherapy agent than unfractionated commercially available GSE, which only enhanced the growth inhibitory effect of 5-FU by 55% (*P*<0.05; compared to 5-FU control). Moreover, we also found that immature F2 (32%) and F3 (35%) were more potent than 5-FU (62% of control value; *P*<0.05) at reducing Caco-2 viability.

Mature seed fractions behaved in a similar manner to immature seed fractions (Figure 3B). Mature seed fractions significantly reduced cell viability (*P*<0.05). F2 and F3 were more cytotoxic to Caco-2 cells (13% and 17% respectively, *P*<0.05) than GSE (47%) (Figure 3B). When cells were exposed to seed fractions and 5-FU, all seed fractions enhanced the capacity of 5-FU to reduce cell viability. GSE significantly enhanced the growth inhibition of 5-FU by 49%. However, F1-F4 significantly enhanced the growth inhibitory effect of 5-FU (62%, 83%, 80% and 60% respectively compared to 5-FU control; *P*<0.05). Moreover, the F2 (13%), F3 (17%) and F4 (50%) fractions were more potent than 5-FU alone (65% of control value; *P*<0.05) (Figure 3B).

## Discussion

Grape seed PCs have been reported to exert health-promoting properties, particularly in the gut [14], [15], [20], [21]. In addition to its anti-cancer efficacy, GSE has been demonstrated to reduce gastrointestinal toxicity following chemotherapy treatment in healthy animals and in normal intestinal cells [15]. Although there is growing evidence to support the chemotherapeutic properties of GSE in colon cancer, identification of the bioactive components responsible remains undefined.

In the current study, PC fractions isolated from commercially available GSE showed different mDPs and molecular sizes. However, these fractions had very low conversion yields (<37%), indicating that less than 40% of the fractions were characterised using the selected analysis techniques, which was unacceptable for this study. The explanation for the low conversion yield in PC fractionsmay have been due to oxidation occurring during generation of GSEs from the winemaking process. Thus, it was decided to isolate PC fractions from fresh grapes collected at different maturities: immature (pre-veraison) and mature (ripe). This decision was based on the observation that immature seeds have a higher conversion yield than mature seeds [22], substantiated by the current study. Moreover, future studies utilizing mass spectrometry are warranted to further define the purity of PCs isolated from grape seeds.

The cytotoxic effects of PC fractions on cancer cells have been reported previously [9], [12], [13]. However, whether these effects occurred through extracellular signalling or following uptake of PCs was not attempted. The present study showed that the most active fractions affecting the viability of Caco-2 cells contained smaller oligomers: F2 and F3. The more potent cytotoxic effects observed in Cabernet Sauvignon seed fractions compared to GSE may have reflected a lower PC yield in GSE (∼24%) compared to PC isolated from fresh grapes (∼60%).

Absorption of PC across cell membranes was greatly dependent upon their mDPs [23], [24]. Only certain lower mDP PCs are absorbed during transit in the gut, leaving the larger mDP PCs (mDP >7) deposited in the gut lumen. This result supports the current data whereby F2 and F3 (mDP 2–6) and likely to be absorbed across the cell membranes were more potent than fractions with a higher mDP, which exert a surface effect only. However, F2 and F3 were more bioactive than F1, possibly due to their higher percentage of galloylation and proportion of epicatechin-3-*O*-gallate as terminal subunits, compared to F1. This result is in agreement with other studies in which PC fractions with higher galloylation were more cytotoxic than PC fractions with lower galloylation when tested on colon cancer cells [9], [25]. However, the actual mechanism of PCs on cell growth remains unknown. Absorbed PC may play a vital role in interfering with cell signalling pathways. PCs have been reported to be inhibitors of androgen receptors in prostate cancer cells[26], [27] and epidermal growth factor receptors on colon cancer cells [28]. In addition, PCs are known to attenuate the PI3-kinase (serine/threonine protein kinase) pathway [14] which could lead to induction of cell cycle arrest at G1 phase [20] and activation of the apoptosis-inducing pathway [29]. Future studies investigating the endpoints of cell growth and apoptosis including PI uptake, caspase 3 activity and sub-G1 DNA content are warranted.

Previous findings have demonstrated that the cytotoxic effects of PCs are dependent on their mDP [9], [13]. Nevertheless, the current study showed that fractions with higher mDP (7–16) failed to exert cytotoxic effects on colon cancer cells. However, the current study used a different solvent system to isolate PC fractions compared to other studies [9], [30]. This solvent system isolates PC fractions with a wider range of molecular mass (619–6063 g/mol) and mDP (2–9) compared to other methods (molecular mass of 552–1232 g/mol, mDP 1–4); implying that the previous studies only measured limited PC fractions (<1200 g/mol) for their biological activities.

The antioxidant capacity of PCs is greatly dependent upon their degree of polymerization and galloylation [9]. The reduction of metal ions, as measured by the FRAP assay, is believed to be positively correlated with the number of hydroxyl groups present in the molecules, and that the points of attachment to transition metal ions in the flavonoid molecules are at the *o*-catechol group of ring B [31]. However, this was not the case in the current study. PC fractions with higher mDP were weaker antioxidants, which is in agreement with previous studies [32], [33]. Fractions with larger subunits tend to self-aggregate and cause stereochemical hindrance [33], [34], thereby exposing fewer hydroxyl groups for radical scavenging activities. Future studies should be performed to address the antioxidant properties of grape seed PCs including the impact on cellular antioxidant status.

The current study was performed based on the basis of a previous study of GSE and chemotherapy on the viability of colon cancer cells [35]. The doses of grape seed fractions in the current study were selected based on the basis of this previous study. 5-FU is an anti-metabolite drugs which block DNA synthesis via inhibition of thymidylate synthase [3].When seed extract fractions were combined with 5-FU, they not only enhanced the impact of 5-FU in killing Caco-2 cells, but also surpassed 5-FU as an anti-cancer agent. In the current study, the effects of PC fractions and 5-FU on Caco-2 cell viability were examined over 24–72 hr. 5-FU (100 uM) significantly reduced cancer cells viability to 60–80%, gastrointestinal toxicity in cancer patients. Exposure of PC fraction and 5-FU to cells for 24–48 hr did show a trend towards a synergistic effect but was not significant (data not shown). Moreover, greater time exposure (72 hr) to PC fractions and 5-FU resulted in greatest reduction of cell viability. The current study also revealed that mature seed fractions were superior to immature seed fractions as chemotherapeutic agents against colon cancer *in vitro*. The chemical profile of the mature seed fractions showed that these effects could be driven by their higher proportion of (−)-epicatechin as terminal subunits compared to the immature seed extract. Another possible explanation could have been the distribution within each fraction of a higher proportion of low molecular weight material in the mature seeds than immature seeds.

The current study showed that the most active fractions affecting the viability of Caco-2 were F2 and F3. However, whether these effects occurred through extracellular signalling or following uptake of PCs is not known. Further studies are warranted to determine the intestinal absorption of PC fractions in normal intestinal cell lines. TheMTT assay is an indirect measure of cell viability based on metabolic activity. The reduction of cell viability observed in the current study could be attributed to an effect on cell cycle or cell proliferation. Hence, additional studies investigating cell cycle, cell proliferation and cell death activities (Annexin V/PI staining, LDL release assay, caspase-3 and Bcl-2 quantification) could provide evidence of the impact of specific sizes of PCs oncell signalling. Testing PC fractions in other colon cell lines (normal and neoplastic) might exclude general toxicity and provide further evidence of PC potential.

In conclusion, further studies are warranted to determine the molecular mechanism of F2 and F3 on cellular pathways associated with colonic neoplasia. Mature seed PC extracts not only have potential uses in human health, but alsoin turn value-add to the wine producing industry, as the mature grape seeds are the major by-products of winemaking. Our data provides compelling evidence that combining PC fractions and 5-FU may be a potential chemopreventative agent in colon cancer treatment regimens; however, further confirmatory *in vitro* studies are required with different classes of chemotherapy drugs followed by *in vivo* cancer model trials to better define the potential role of grape seed PCs against colon cancer.
